# Supplementary material for: Pitavastatin and Ivermectin Enhance the Efficacy of Paclitaxel in Chemoresistant High-Grade Serous Carcinoma
Source: Cancers (Basel). 2022 Sep 7;14(18):4357. doi: 10.3390/cancers14184357 (PMC9496819; doi:10.3390/cancers14184357)
Supplement: Supplementary file 1 [file cancers-14-04357-s001.zip › cancers-1884687-supplementary.pdf]

Supplementary Material

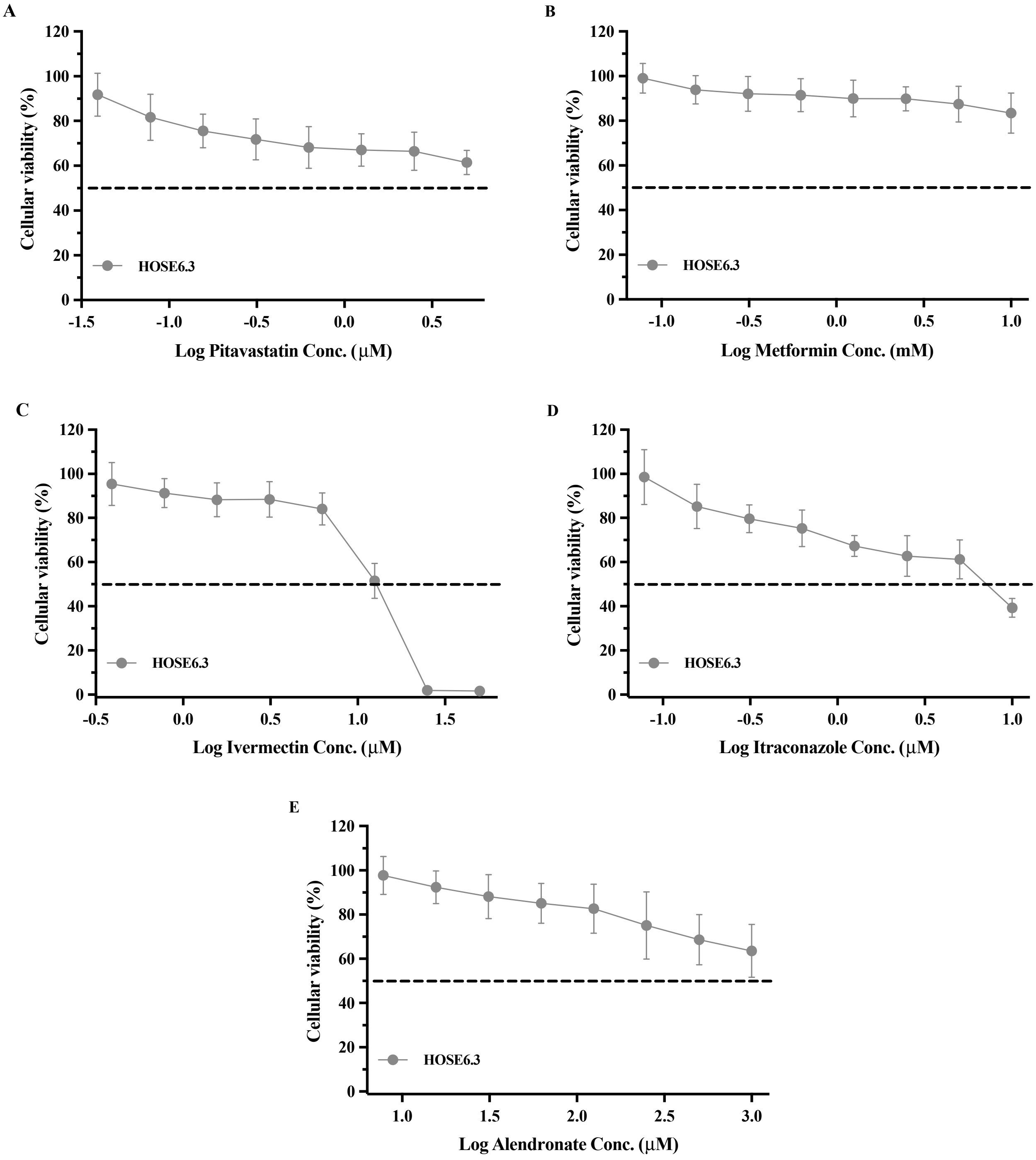

Figure S1. Repurposed drugs demonstrate low efficacy in reducing cellular viability of HOSE6.3 cells. (**A** – **E**) Dose-response curves for HOSE6.3 cells obtained by Presto Blue assay after exposure to increasing concentrations of (**A**) Pitavastatin (0.04 to 5 μM), (**B**) Metformin (0.08 to 10 mM), (**C**) Ivermectin (0.39 to 50 μM), (**D**) Itraconazole (0.39 to 50 μM) and (**E**) Alendronate (7.81 to 1000 μM) for 48 h. IC_{50} values are represented by a dot line in each dose-response curve. All assays were done in triplicates in at least three independent experiments. Data are expressed as mean ± standard deviation and plotted using GraphPad Prism Software Inc. v8. Scale bar, 20 mm.

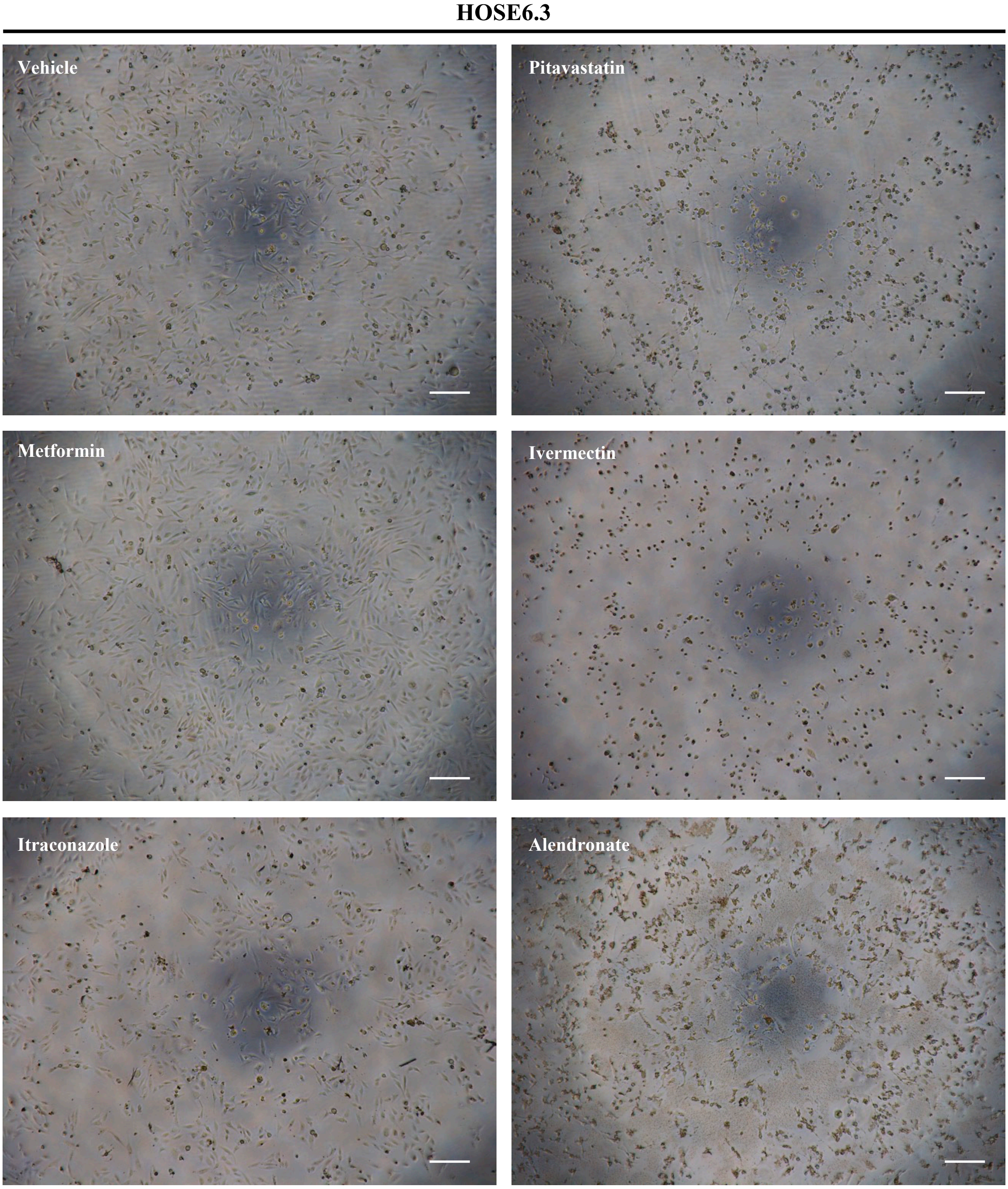

Figure S2. Representative microscopy images of HOSE6.3 cells, after exposure to vehicle, Pitavastatin, Metformin, Ivermectin, Itraconazole and Alendronate at the highest concentration used for each drug for 48 h. All assays were done in triplicates in at least three independent experiments. Scale bar, 20 µm.

Table S1. Cellular viability (%) of OVCAR8 and OVCAR8 PTX R P cells, after exposure to a fixed dose-ratio that correspond to 0.25, 0.5, 1, 2 and 4 times the individual IC_{50} values of each drug for 48 h. The combined treatment was co-administered at the same time. All assays were done in triplicates in at least three independent experiments.

Treatment	Concentration	OVCAR8		OVCAR8 PTX R P	
		Cellular viability (%)		Cellular viability (%)	
		Media	SD	Media	SD
Paclitaxel (nM)	0.25 x IC50	80.757	5.416	90.528	2.353
	0.5 x IC50	78.038	8.142	87.466	4.860
	IC50	53.946	3.312	86.708	4.216
	2 x IC50	29.671	3.816	80.894	7.527
	4 x IC50	12.151	2.665	74.763	1.916
Pitavasvatin (µM)	0.25 x IC50	61.378	8.811	59.998	1.774
	0.5 x IC50	51.912	13.448	50.426	1.186
	IC50	45.624	4.912	45.406	2.741
	2 x IC50	27.970	6.045	28.330	5.862
	4 x IC50	26.234	10.455	26.234	10.455
Paclitaxel (nM) + Pitavastatin (µM)	0.25 x IC50	53.275	7.058	57.336	8.149
	0.5 x IC50	37.656	0.811	36.095	6.457
	IC50	19.100	2.025	24.091	0.683
	2 x IC50	14.958	3.148	15.104	3.044
	4 x IC50	10.729	0.024	11.373	0.620
Metformin (mM)	0.25 x IC50	72.126	1.341	68.105	9.000
	0.5 x IC50	63.520	3.097	59.114	9.045
	IC50	44.207	8.087	44.548	6.851
	2 x IC50	16.568	6.162	14.871	5.490
	4 x IC50	6.948	1.168	12.397	4.071
Paclitaxel (nM) + Metformin (mM)	0.25 x IC50	60.893	14.666	70.029	8.434
	0.5 x IC50	46.734	6.629	57.874	7.065
	IC50	21.111	2.909	26.557	4.411
	2 x IC50	14.153	4.135	14.428	2.235
	4 x IC50	5.543	0.951	9.163	2.125
Ivermectin (µM)	0.25 x IC50	77.004	5.400	81.152	4.877
	0.5 x IC50	69.934	3.792	65.026	10.605
	IC50	46.412	1.176	47.496	1.382
	2 x IC50	0.759	0.072	0.966	0.072
	4 x IC50	0.867	0.140	1.370	0.394
Paclitaxel (nM) + Ivermectin (µM)	0.25 x IC50	69.955	6.855	80.717	3.183
	0.5 x IC50	44.541	3.217	45.360	8.110
	IC50	8.706	2.890	6.195	1.862
	2 x IC50	0.820	0.320	1.048	0.422
	4 x IC50	0.613	0.438	0.865	0.348
Itraconazole (µM)	0.25 x IC50	92.297	10.540	92.844	5.230
	0.5 x IC50	99.490	8.443	97.871	6.224
	IC50	102.809	12.788	95.181	4.623
	2 x IC50	101.169	7.059	87.752	8.305
	4 x IC50	86.903	10.831	73.512	11.071
Paclitaxel (nM) + Itraconazole (µM)	0.25 x IC50	83.754	7.372	95.238	7.076
	0.5 x IC50	68.477	6.173	83.660	4.073
	IC50	45.331	7.790	56.556	3.372
	2 x IC50	31.682	11.529	34.842	5.969
	4 x IC50	22.879	8.467	25.781	5.294
Alendronate (µM)	0.25 x IC50	103.500	14.967	94.205	1.914
	0.5 x IC50	101.117	13.794	84.877	5.677
	IC50	53.983	1.035	43.609	3.973
	2 x IC50	32.912	5.431	32.275	7.048
	4 x IC50	30.497	9.853	30.685	4.090
Paclitaxel (nM) + Alendronate (µM)	0.25 x IC50	86.901	10.541	88.880	11.047
	0.5 x IC50	83.410	8.147	65.998	4.907
	IC50	16.141	4.990	17.529	1.584
	2 x IC50	9.314	7.929	14.731	2.316
	4 x IC50	9.233	7.489	11.088	3.682

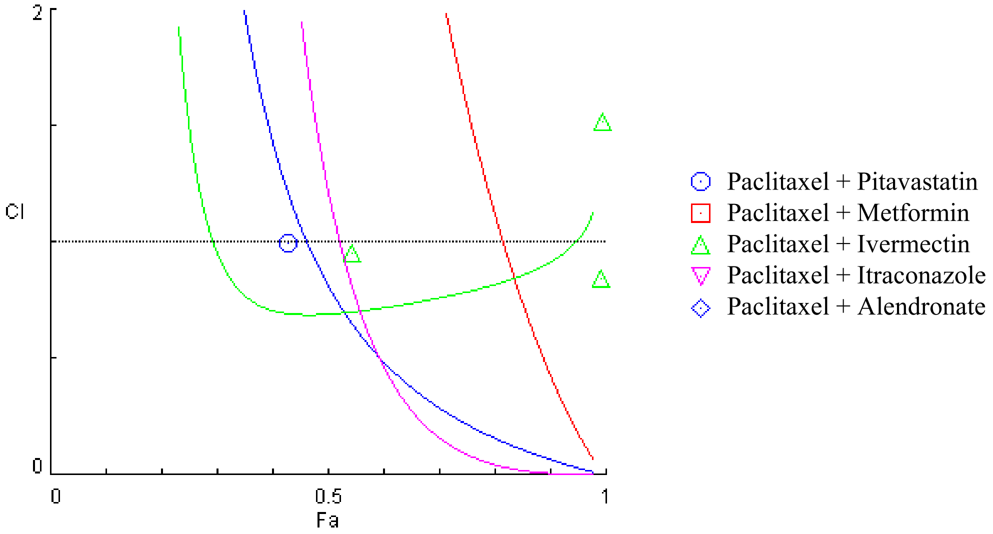

Figure S3. Combining Paclitaxel with repurposed drugs has an antagonistic effect on HOSE6.3 cells. Chou-Talalay method effect level (Fa) – Combinatory Index (CI) plot showing drug synergism of HOPSE6.3 cells, after exposure to a fixed dose-ratio that correspond to 0.25, 0.5, 1, 2 and 4 times the individual IC_{50} values each drug, e.g., Paclitaxel combined with Pitavastatin, Metformin, Ivermectin, Itraconazole and Alendronate for 48 h. The combined treatment was co-administered at the same time. All assays were done in triplicates in at least three independent experiments. CI was plotted on the y-axis as a function of Fa on the x-axis to evaluate drug synergism. CI: <1 (synergism), =1 (additivity) and >1 (antagonism).

Table S2. Fractional effect (Fa) values and respective combinatory index (CI) values showing drug synergism of HOSE6.3 cells, after exposure to a fixed dose-ratio that correspond to 0.25, 0.5, 1, 2 and 4 times the individual IC_{50} values of each drug for 48 h. The combined treatment was co-administered at the same time. All assays were done in triplicates in at least three independent experiments. CI: >1 (antagonism), =1 (additivity) and <1 (synergism).

				HOSE6.3	
**Combination** (Drug 1 + Drug 2)	**Total Dose** (Drug 1 + Drug 2)	**Dose** (Drug 1)	**Dose** (Drug 2)	**Fa**	**CI Value**
**Paclitaxel (nM) + Pitavastatin (µM)**	2.7	2.5	0.2	0.430	0.994
	5.4	5	0.4	0.271	4.777
	10.8	10	0.8	0.234	4.675
	21.6	20	1.6	0.146	18.988
	43.2	40	3.2	0.115	26.816
**Paclitaxel (nM) + Metformin (mM)**	2.875	2.5	0.375	0.181	39.581
	5.75	5	0.750	0.156	41.167
	11.5	10	1.5	0.132	45.791
	23	20	3	0.094	109.733
	46	40	6	0.083	101.352
**Paclitaxel (nM) + Ivermectin (µM)**	6.25	2.5	3.75	0.995	1.515
	12.5	5	7.5	0.991	0.842
	25	10	15	0.544	0.956
	50	20	30	0.110	54.256
	100	40	60	0.093	57.939
**Paclitaxel (nM) + Itraconazole (µM)**	6.25	2.5	3.75	0.155	91.946
	12.5	5	7.5	0.137	83.464
	25	10	15	0.102	166.235
	50	20	30	0.048	2388.570
	100	40	60	0.041	2364.560
**Paclitaxel (nM) + Alendronate (µM)**	40	2.5	37.5	0.084	1457.650
	80	5	75	0.031	54522.200
	160	10	150	0.030	31314.4
	320	20	300	0.030	15657.200
	640	40	600	0.001	1.00E10

Table S3. Cellular viability (%) of HOSE6.3 cells, after exposure to a fixed dose-ratio that correspond to 0.25, 0.5, 1, 2 and 4 times the individual IC_{50} values of each drug for 48 h. The combined treatment was co-administered at the same time. All assays were done in triplicates in at least three independent experiments. SD, standard deviation.

Treatment	Concentration	HOSE6.3 Cellular viability (%) Media	SD
**Paclitaxel (nM)**	0.25 x IC50	78.854	5.925
	0.5 x IC50	75.466	6.905
	IC50	70.822	6.979
	2 x IC50	68.366	5.776
	4 x IC50	65.718	7.079
**Pitavasvatin (µM)**	0.25 x IC50	81.849	7.681
	0.5 x IC50	76.512	7.815
	IC50	68.462	3.472
	2 x IC50	65.888	5.045
	4 x IC50	61.442	2.761
**Paclitaxel (nM) + Pitavastatin (µM)**	0.25 x IC50	88.488	5.102
	0.5 x IC50	85.399	5.346
	IC50	76.611	4.971
	2 x IC50	72.886	4.878
	4 x IC50	57.037	6.338
**Metformin (mM)**	0.25 x IC50	96.286	4.707
	0.5 x IC50	97.224	1.001
	IC50	95.033	1.429
	2 x IC50	96.032	1.778
	4 x IC50	92.366	2.639
**Paclitaxel (nM) + Metformin (mM)**	0.25 x IC50	91.847	6.149
	0.5 x IC50	90.569	7.830
	IC50	86.845	5.504
	2 x IC50	83.700	2.788
	4 x IC50	81.860	2.706
**Ivermectin (µM)**	0.25 x IC50	100.990	2.144
	0.5 x IC50	99.694	3.725
	IC50	53.462	2.873
	2 x IC50	0.539	1.365
	4 x IC50	0.000	0.544
**Paclitaxel (nM) + Ivermectin (µM)**	0.25 x IC50	90.745	5.112
	0.5 x IC50	89.023	4.590
	IC50	45.582	3.931
	2 x IC50	0.922	1.661
	4 x IC50	0.457	0.938
**Itraconazole (µM)**	0.25 x IC50	86.353	3.252
	0.5 x IC50	84.356	1.857
	IC50	84.735	3.865
	2 x IC50	79.729	1.179
	4 x IC50	77.370	3.174
**Paclitaxel (nM) + Itraconazole (µM)**	0.25 x IC50	95.856	1.914
	0.5 x IC50	95.195	1.303
	IC50	89.804	3.721
	2 x IC50	86.268	3.661
	4 x IC50	84.498	3.203
**Alendronate (µM)**	0.25 x IC50	101.792	6.109
	0.5 x IC50	101.959	6.966
	IC50	92.147	2.125
	2 x IC50	89.188	5.221
	4 x IC50	84.305	5.970
**Paclitaxel (nM) + Alendronate (µM)**	0.25 x IC50	100.808	2.093
	0.5 x IC50	96.984	1.927
	IC50	96.973	0.865
	2 x IC50	96.946	0.508
	4 x IC50	91.567	1.748
